# Increase of aerobic glycolysis mediated by activated T helper cells drives synovial fibroblasts towards an inflammatory phenotype: new targets for therapy?

**DOI:** 10.1186/s13075-021-02437-7

**Published:** 2021-02-15

**Authors:** Peter Kvacskay, Nina Yao, Jürgen-Heinz Schnotz, Roberta Scarpone, Rui de Albuquerque Carvalho, Karel D. Klika, Wolfgang Merkt, Theresa Tretter, Hanns-Martin Lorenz, Lars-Oliver Tykocinski

**Affiliations:** 1 Department of Medicine V, Division of Rheumatology, University of Heidelberg, INF 410, 69120 Heidelberg, Germany; 2 Department of Life Sciences, Faculty of Sciences and Technology, University of Coimbra, Coimbra, Portugal; 3 REQUIMTE/LAQV, Group of Pharmaceutical Technology, Faculty of Pharmacy, University of Coimbra, Coimbra, Portugal; 4 Molecular Structure Analysis, German Cancer Research Center (DKFZ), Heidelberg, Germany

**Keywords:** Rheumatoid arthritis, Synovial fibroblasts, Fibroblast-like synoviocytes, T lymphocytes, T helper cells, Metabolism, Glycolysis, Janus kinases, Inflammation, Translational research

## Abstract

**Background:**

A dysregulated glucose metabolism in synovial fibroblasts (SF) has been associated with their aggressive phenotype in rheumatoid arthritis (RA). Even though T helper (Th) cells are key effector cells in the propagation and exacerbation of synovitis in RA, little is known about their influence on the metabolism of SF. Thus, this study investigates the effect of Th cells on the glucose metabolism and phenotype of SF and how this is influenced by the blockade of cytokines, janus kinases (JAKs) and glycolysis.

**Methods:**

SF from patients with RA or osteoarthritis (OA) were cultured in the presence of a stable glucose isotopomer ([U-^13^C]-glucose) and stimulated with the conditioned media of activated Th cells (ThCM). Glucose consumption and lactate production were measured by proton nuclear magnetic resonance (^1^H NMR) spectroscopy. Cytokine secretion was quantified by ELISA. The expression of glycolytic enzymes was analysed by PCR, western blot and immunofluorescence. JAKs were blocked using either baricitinib or tofacitinib and glycolysis by using either 3-bromopyruvate or FX11.

**Results:**

Quiescent RASF produced significantly higher levels of lactate, interleukin (IL)-6 and matrix metalloproteinase (MMP) 3 than OASF. Stimulation by ThCM clearly changed the metabolic profile of both RASF and OASF by inducing a shift towards aerobic glycolysis with strongly increased lactate production together with a rise in IL-6 and MMP3 secretion. Interestingly, chronic stimulation of OASF by ThCM triggered an inflammatory phenotype with significantly increased glycolytic activity compared to unstimulated, singly stimulated or re-stimulated OASF. Finally, in contrast to cytokine-neutralizing biologics, inhibition of JAKs or glycolytic enzymes both significantly reduced lactate production and cytokine secretion by Th cell-stimulated SF.

**Conclusions:**

Soluble mediators released by Th cells drive SF towards a glycolytic and pro-inflammatory phenotype. Targeting of JAKs or glycolytic enzymes both potently modulate SF’s glucose metabolism and decrease the release of IL-6 and MMP3. Thus, manipulation of glycolytic pathways could represent a new therapeutic strategy to decrease the pro-inflammatory phenotype of SF.

**Supplementary Information:**

The online version contains supplementary material available at 10.1186/s13075-021-02437-7.

## Background

Rheumatoid arthritis (RA) is a systemic autoimmune disease characterized by chronic inflammation, recurring synovitis and destruction of the cartilage and bone [1, 2]. The characteristic transformation of the synovial membrane into a destructive tumour-like pannus is accompanied by a persistent intra-articular invasion of immune cells. Synovial fibroblasts (SF), also known as fibroblast-like synoviocytes, are the major cell type within the hyperplastic pannus and the main effector cells of joint destruction. SF of RA patients (RASF) present an aggressive phenotype with abnormally increased proliferation, secretion of pro-inflammatory cytokines and tissue-invasive properties [3–5]. The aberrant fibroblast phenotype and the hypoxic and nutrient-deprived micro-environment of the pannus are characteristics which are also found in solid tumours. For tumour cells, it is well known that they adapt their glucose metabolism to meet the increased bioenergetic and biosynthetic demands in a tumour micro-environment [6, 7]. Already in 1924, Warburg showed that malignant cells produce significantly higher amounts of lactate than normal cells under normoxic conditions [8, 9]. He stated that upregulation of aerobic glycolysis allowed malignant cells to survive the hypoxic conditions prevailing in highly proliferative tumour tissues.

There is growing evidence lately that this well-described Warburg effect, i.e. the metabolic switch from oxidative phosphorylation towards glycolysis, is also a characteristic feature of inflamed joints of RA patients [10, 11]. Several studies have demonstrated that lactate levels were significantly increased while glucose levels were decreased in synovial fluid or in synovial tissue of RA patients compared to those of osteoarthritis (OA) patients or healthy individuals [12–15]. In the serum of RA patients, an increase of both glucose and lactate levels has been described [16]. Using fluorodeoxyglucose-positron emission tomography, the increased glucose uptake by cells of the pannus was able to be imaged, suggesting an enhanced glycolytic activity of RASF and invading immune cells [17, 18]. Moreover, a significant correlation between mitochondrial dysfunction, enhanced aerobic glycolysis and the inflammatory, destructive properties of RASF has been described [19–23].

Although a dysregulated glucose metabolism in RASF has been suggested to play a critical role in the pathogenesis of RA, very little is known about the impact of activated immune cells on the regulation of the glucose metabolism of SF. The association of RA, especially in the most erosive form, with specific HLA-DR alleles provides strong evidence for a key role of T helper (Th) cells in RA pathogenesis and disease severity. T lymphocytes infiltrate the joints of RA patients and constitute about 30–50% of all cell types in the sub-lining region of synovial tissues. In this way, T cells and SF are in close contact and stimulate each other by direct cell-cell contact or by the release of soluble factors [24]. The interaction between Th17 cells and SF has been described as a key mechanism for the development of synovial tissue inflammation [25]. Interaction and reciprocal activation of Th cells and RASF have recently been shown to induce and amplify inflammatory responses and to result in a metabolic shift towards glycolysis in SF to meet the increased metabolic demand [26]. On the other hand, interaction with Th cells induces immuno-suppressive functions of SF, a capacity that has been shown to be reduced in RASF compared to OASF [27, 28]. Altogether, cross-talk with Th cells clearly affects SF’s phenotype and function.

In this study, we investigated the impact of Th cells on the glucose metabolism and phenotype of SF. Further, we compared the effect of chronic stimulation with single stimulation and re-stimulation by Th cells on SF. Finally, we analysed the potency of cytokine-neutralizing biologics and janus kinase inhibitors (JAKi), used in the treatment of inflammatory rheumatic diseases like RA, as well as of inhibitors targeting glycolytic enzymes in limiting the T cell-mediated induction of a glycolytic and inflammatory phenotype in SF. OASF have been described to differ from RASF in many ways, e.g. OASF are less resistant to apoptosis, have a different epigenetic signature and do not possess an intrinsically activated, aggressive and invasive phenotype like RASF [29–31]. In the present study, OASF were used as a degenerative disease control to RASF.

## Methods

### Cell isolation and culture

SF from patients with either OA or RA were isolated from synovial tissues collected during diagnostic arthroscopy or therapeutic joint surgery as described previously [28]. CD4^+^ Th cells were isolated from heparinized venous blood of RA patients or normal healthy donors (NHD) by density gradient centrifugation and separation using the MojoSort human CD4 T cell isolation kit (BioLegend) according to the manufacturer’s instructions (purity ≥ 98%). All RA patients fulfilled the American College of Rheumatology/European League Against Rheumatism criteria for the classification of RA [32].

SF were cultured in DMEM-F12 medium (Merck) supplied with 10% heat-inactivated foetal calf serum (Thermo Fisher Scientific). Passages 4–10 were used for experiments. Th cells were stimulated with anti-CD3 and anti-CD28 (both 1 μg/ml, Thermo Fisher Scientific) in glucose-free RPMI-1640 medium (Biological Industries) supplemented with 10% foetal calf serum and the stable glucose isotope tracer [U-^13^C]-glucose (2 g/l, Tracer tec). Th cell-conditioned media (ThCM) were collected on day 4. For most experiments, SF were stimulated with ThCM diluted 1:5 in RPMI-1640 medium. Hypoxic culture conditions were maintained using a glove box (Coylab) and an incubator (Heracell 150i, Thermo Fisher Scientific) with oxygen level control. In some experiments, SF were stimulated with different concentrations of recombinant human interleukin (IL)-1β, IL-17A (both BioLegend), interferon (IFN)γ (R&D Systems) or tumour necrosis factor (TNF)α (Peprotech). Cytokines or cytokine receptors were neutralized using canakinumab (Novartis), etanercept (Pfizer), secukinumab (Novartis) or tocilizumab (Roche). Janus kinases (JAKs) were targeted by baricitinib (TargetMol) or tofacitinib (Pfizer). Hexokinase (HK) 2 was inhibited by 3-bromopyruvate (3-BrPa) (Gentaur) and lactate dehydrogenase (LDH)-A by FX11 (Merck).

Cell survival was determined by Annexin V and propidium iodide staining and measured by flow cytometry using a BD FACSCanto II analyser (BD Biosciences). Staurosporin-treated cells (2 μM, 24 h) served as a positive control.

### Analysis of lactate and glucose levels by ^1^H NMR spectroscopy

SF were cultured in the presence of [U-^13^C]-glucose either with or without stimulation by ThCM. Culture supernatants were harvested on day 4 and analysed using a Bruker Avance II spectrometer equipped with a 5-mm indirect detection probe (Bruker BioSpin) operating at 600 MHz for ^1^H. The ^1^H NMR spectra with water suppression using presaturation were acquired with 43k points defining a spectral width of 7.2 kHz using a 30° radiofrequency pulse and a total repetition time of 10 s to ensure full relaxation of the ^1^H nuclei. Spectral analyses were performed using NUTSpro™ NMR software (Acorn NMR Inc.). Each free induction decay was multiplied by a decaying exponential with a decay constant of 0.2 Hz prior to Fourier transformation. For quantification, sodium fumarate (10 mM) dissolved in a 0.2-M phosphate buffer solution prepared with D_2_O (99.9%) was used as an internal standard.

### Quantification of cytokine secretion

The concentrations of IL-6, IL-8 and matrix metalloproteinase (MMP) 3 in culture supernatants were quantified by enzyme-linked immunosorbent assay (ELISA) using the Duo Set ELISA kits (R&D Systems) for all cytokines according to the manufacturer’s instructions. Cytokines in the ThCM were quantified using the LEGENDplex Human Th Cytokine Panel 12-plex assay kit (BioLegend).

### In vitro scratch migration assay

To assess the migration of SF in the presence or absence of stimulation by soluble mediators released by activated Th cells or recombinant human TNFα, IL-17A or IFNγ (10 ng/ml each, all PeproTech), we performed an in vitro scratch assay as described [33].

### RNA isolation, cDNA transcription and quantitative RT-PCR

The High Pure RNA isolation kit (Roche) was used to isolate total RNA. Total RNA was reverse transcribed into cDNA with the QuantiTect reverse transcription kit (Qiagen). RT-PCRs were performed using the PowerUp SYBR Green master mix and a StepOnePlus system (both Applied Biosystems). The following primers were utilized: *HK2*—Fwd-AAGGCTTCAAGGCATCTG, rev-CCACAGGTCATCATAGTTCC; *PFKp*—Fwd-AGATCCATAAGGAGGCCGTG, rev-AGAACGAAGGTCCTCTGGTG; *PKM2*—Fwd-ATTATTTGAGGAACTCCGCCGCCT, rev-ATTCCGGGTCACAGCAATGATGG; and *LDH-A*—Fwd-ACCCAGTTTCCACCATGATT, rev-CCCAAAATGCAAGGAACACT.

### Western blot analysis

Isolation of total cell extracts from SF was performed by lysing cells in RIPA buffer (ccpro) containing a protease inhibitor cocktail (completeMini, Boehringer) for 20 min on ice. After dilution in a loading buffer, protein samples were resolved using standard SDS-PAGE and transferred onto nitrocellulose membranes. HK2, phosphofructokinase (PFK)p, pyruvate kinase M2 (PKM2) and LDH-A were detected by corresponding antibodies (CellSignaling). β-Actin was used as a loading control. Quantification was performed by densiometric analysis using ImageJ2.

### Fluorescence microscopy

SF were left to adhere on glass coverslips and were either stimulated with ThCM for 4 days or left unstimulated. Cells were fixed by paraformaldehyde and permeabilized with ethanol. Fluorescent antibody staining was performed using antibodies against HK2 (eBioscience), PKM2 (CellSignaling) and Cy3-labelled anti-rabbit-IgG. Cell nuclei were stained with 4′,6-diamidino-2-phenylindole (DAPI). The analysis was performed at the Imaging Facility of the Center for Molecular Biology Heidelberg (ZMBH) using an Olympus IX81 microscope.

### Statistics

Results are presented as the mean ± SEM. The Mann-Whitney *U* test and the Wilcoxon signed-rank test were applied to unpaired and paired, respectively, sample sets using GraphPad Prism for statistical analysis. Values of *p* less than 0.05 were considered statistically significant.

## Results

### Activated Th cells release mediators that induce a metabolic shift towards aerobic glycolysis in SF

**Fig. 1:**
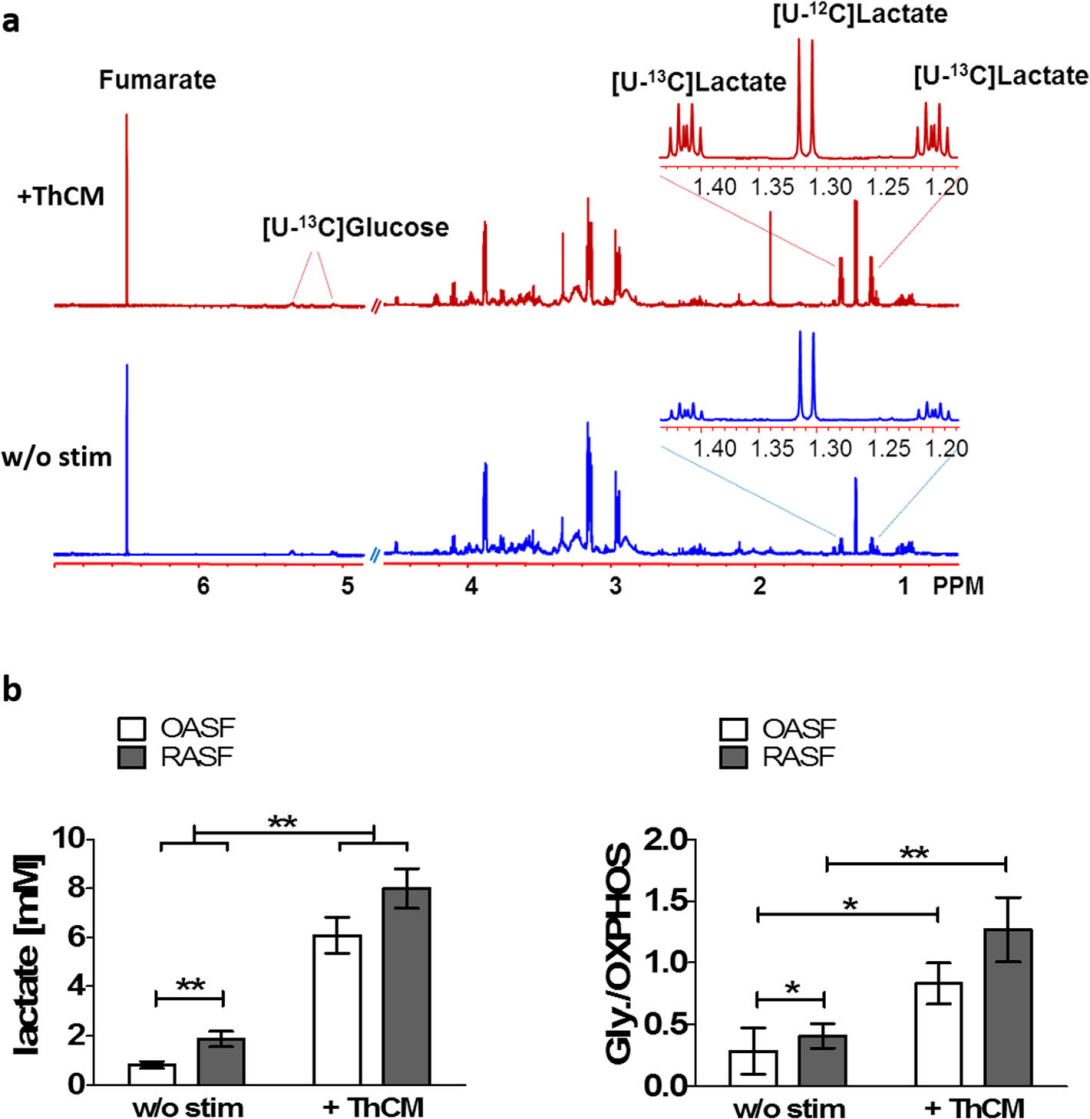
Th cells induce a metabolic shift towards aerobic glycolysis in SF. OASF (*n* = 12) and RASF (*n* = 12) were cultured for 4 days in the presence of [U-^13^C]-glucose either under resting conditions or stimulated by conditioned culture media of activated Th cells (ThCM). The amount of [U-^13^C]-glucose and [U-^13^C]-lactate produced by the SF was measured using ^1^H NMR spectroscopy. **a** Representative examples of ^1^H NMR spectra of SF stimulated by ThCM (red) and unstimulated SF (blue). **b** The amount of secreted [U-^13^C]-lactate and the ratio of [U-^13^C]-glucose metabolized by glycolysis (Gly) and those metabolized by oxidative phosphorylation (OXPHOS) was determined for SF culture supernatants. Results are presented as the mean ± SEM. *p < 0.05, **p < 0.01, Mann-Whitney *U* test and Wilcoxon signed-rank test. w/o stim, without stimulation

In order to investigate the influence of Th cells on the glucose metabolism of both OASF and RASF, SF were cultured in the presence of a stable glucose isotope ([U-^13^C]-glucose) either under resting conditions or stimulated by ThCM. The amount of [U-13C]-glucose remaining and the glucose-derived [U-^13^C]-lactate produced by the SF was measured using ^1^H NMR spectroscopy. This method allowed us to quantify the concentration of residual, non-metabolized glucose in the culture supernatants as well as the concentration of generated and secreted lactate as the product of glycolysis. By calculating the amount of glucose that was consumed by SF, but not metabolized into lactate, it was possible to indirectly quantify the level of oxidative glucose metabolism. Figure 1a shows representative ^1^H NMR spectra of culture supernatants from unstimulated SF and from SF stimulated by ThCM. Stimulation with ThCM resulted in a decrease of glucose and a clear increase of lactate in SF culture supernatants. Comparing OASF and RASF under resting conditions, RASF showed significantly more lactate production and a significantly higher ratio of glucose metabolized by aerobic glycolysis versus that metabolized by oxidative phosphorylation than OASF (Fig. 1b). Stimulation by conditioned culture medium from Th cells of RA patients resulted in a significant increase in lactate production and a significant shift towards glycolytic glucose metabolism by both RASF and OASF (Fig. 1b). However, in contrast to resting conditions, no significant difference in lactate production was detected between RASF and OASF under stimulation with ThCM, although a trend towards still higher lactate levels and glycolytic rates in RASF could be seen (Fig. 1b). Similar to the stimulation by RA patients’ ThCM, stimulation of OASF and RASF by conditioned culture medium from Th cells of healthy donors resulted in a strong increase of lactate production by SF (Additional file 1: Fig. S1). Since there were no significant detectable differences between the induction of glycolysis in SF by ThCM from the T cells of healthy individuals or RA patients, all of the experiments in this study were carried out with stimulation of SF by RA patients’ ThCM. As shown in Additional file 2: Fig. S2, the stimulatory effect of ThCM on SF’s lactate production rates was dose-dependent.

Analysis of the cytokines present in the ThCM used to stimulate the SF revealed that IFNγ represented the highest content of all the cytokines tested (Additional file 3: Fig. S3). Additionally, TNFα, IL-6, IL-9, IL-22, IL-5, IL-13 and to a lesser extent IL-17A and IL-10 were also detected. However, at 151.07 ± 36.42 ng/ml, the concentration of IFNγ was more than 25-fold higher than any of the other cytokines (Additional file 3: Fig. S3).

Since hypoxia has been described as a characteristic micro-environmental feature of inflamed joints of RA patients with a mean ambient oxygen tension of only 3.2% O_2_ [34], we therefore next investigated the influence of hypoxic conditions on the glucose metabolism of SF. As expected, culture under hypoxic conditions (3% O_2_) resulted in an increased lactate production by both OASF and RASF. Similar to what had been observed under normoxic conditions, RASF showed significantly higher lactate production than OASF under hypoxia in the absence of further stimulation (Additional file 4: Fig. S4). Stimulation by ThCM under hypoxic conditions equally induced a significant and dose-dependent upregulation of lactate production in both RASF and OASF. However, a further shift from oxidative towards glycolytic glucose metabolism could not be detected in stimulated versus un-stimulated SF under hypoxia (Additional file 4: Fig. S4). Together, these results demonstrate that RASF displayed a higher basic rate of glycolysis under resting conditions compared to OASF; but under stimulation by Th cells, OASF acquired a similar high glycolytic activity as RASF. Moreover, the data indicate that particularly RASF use glycolysis as a means of energy production under hypoxic conditions.

### Soluble mediators released by activated Th cells induce a pro-inflammatory phenotype in SF

**Fig. 2:**
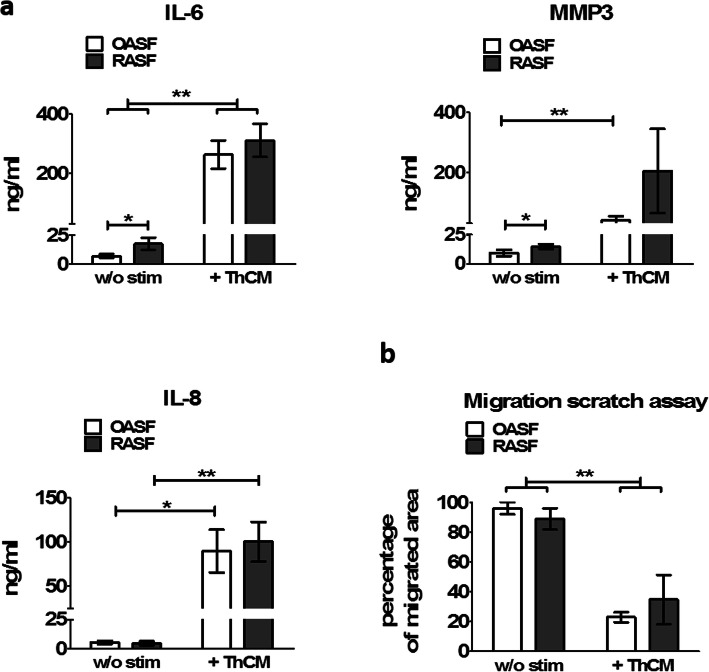
Mediators released by Th cells induce pro-inflammatory cytokine expression by SF but diminish their migration. Secretion of pro-inflammatory cytokines correlates with enhanced glycolytic activity in SF. OASF and RASF were cultured in the presence or absence of ThCM. **a** After 4 days, the concentration of interleukin (IL)-6, IL-8, and matrix metalloprotease (MMP)3 within the culture supernatants was quantified by ELISA (*n* = 10). **b** An in vitro scratch assay was performed to investigate the cell migration of SF cultured with or without ThCM (*n* = 6). Data are presented as the mean ± SEM. *p < 0.05, **p < 0.01, Mann-Whitney *U* test and Wilcoxon signed-rank test

To determine whether activated Th cells also stimulated the development of a pro-inflammatory profile in SF, we analysed the secretion of IL-6, IL-8 and MMP3 by OASF and RASF cultured under resting conditions or under stimulation by ThCM. In the absence of stimulation, RASF showed significantly higher levels of IL-6 and MMP3 secretion compared to OASF (Fig. 2a). Stimulation with ThCM strongly augmented the secretion of IL-6, IL-8 and MMP3 in both groups. Cytokines IL-6 and IL-8 can also be secreted by activated Th cells, however only in much lower amounts compared to Th cell-stimulated SF as reported previously [27, 28] and shown in Additional file 3: Fig. S3. The ThCM used here to stimulate SF only contained approximately 6 ng/ml IL-6 (6.04 ± 2.95 ng/ml). Therefore, the proportion of Th cell-derived IL-6 and IL-8 in the supernatants of ThCM-stimulated SF can be neglected.

To investigate the impact of stimulation by Th cells on the migration of SF, we performed an in vitro scratch assay. No difference in the migratory capacities could be detected between OASF and RASF under resting conditions. Interestingly, under stimulation by ThCM, both RASF and OASF displayed significantly decreased migration rates compared to unstimulated conditions (Fig. 2b and Additional file 5: Fig. S5). Similarly, stimulation of SF with a combination of IFNγ, TNFα and IL-17A also strongly reduced their migration. This effect was less pronounced when the SF were stimulated by individual cytokines alone (Additional file 5: Fig. S5). Thus, mediators released by activated Th cells induced a strong metabolic shift towards glycolysis in SF in tandem with an augmented secretion of inflammatory mediators and reduced migratory properties.

### Chronic stimulation by Th cells triggers a highly glycolytic and inflammatory phenotype in OASF

**Fig. 3:**
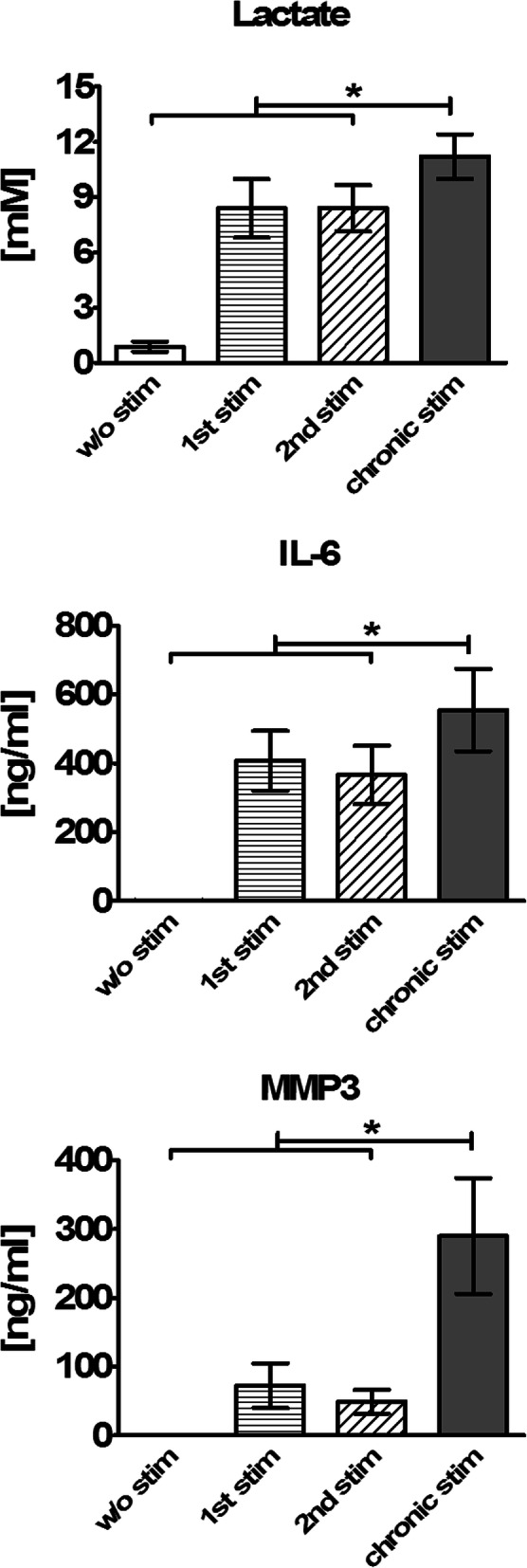
Chronic stimulation by Th cells triggers a glycolytic and pro-inflammatory phenotype in OASF. OASF were cultured for a total period of 18 days under four different conditions: The first group was cultured without stimulation (w/o stim). The second group was stimulated on d14 of culture (1st stim). The third group was first stimulated between d1 and d4, then remained unstimulated until a second stimulation on d14 (2nd stim). The fourth group was repeatedly stimulated between d1 and d12 and re-stimulated on d14 (chronic stim). On d18, the concentrations of lactate, IL-6 and MMP3 were quantified for all four groups (*n* = 7). Results are presented as the mean ± SEM. Statistical significances between the chronically stimulated group and the unstimulated, the singly stimulated and the re-stimulated group are shown. *p < 0.05, Wilcoxon signed-rank test

Fibroblasts have been described to play a critical role in the induction of a chronic persistent inflammation in RA, and stimulation seems to prime even SF from non-inflamed joints for a pro-inflammatory memory response [35]. Here, we wanted to investigate whether a single stimulation or a chronic stimulation by Th cells primes the phenotype and glucose metabolism of OASF. Therefore, OASF were cultured under four different conditions: The first group was cultured without stimulation for a total period of 18 days (unstimulated). The second group of OASF was stimulated by ThCM on day 14 of culture (primary stimulation (1st stim)). The third group was first stimulated between days 1 and 4 of culture, then washed and remained unstimulated until a second stimulation on day 14 (re-stimulation (2nd stim)). And finally, the fourth group was repeatedly stimulated between days 1 and 12 and re-stimulated on day 14 by ThCM (chronic stimulation). On day 18, we quantified the concentrations of lactate, IL-6 and MMP3 in the culture supernatants of all four groups. As presented in Fig. 3, unstimulated SF showed very low lactate production and nearly no IL-6 or MMP3 secretion. Single stimulation on day 14 resulted in a sharp increase of lactate and IL-6 production and a mild upregulation of MMP3 secretion. Remarkably, pre-stimulation and later re-stimulation of SF did not induce a memory response with higher lactate, IL-6 or MMP3 production compared to the singly stimulated group. However, chronic stimulation of the SF resulted in a significantly increased secretion of lactate, as well as of IL-6 and MMP3 (Fig. 3). Hence, a repeated stimulation of OASF by Th cells, but not a single re-stimulation, triggered an aggressive phenotype with significantly higher production of lactate and inflammatory cytokines compared to once only stimulated OASF.

### Stimulation of SF by Th cells enhance the expression of glycolytic enzymes

**Fig. 4:**
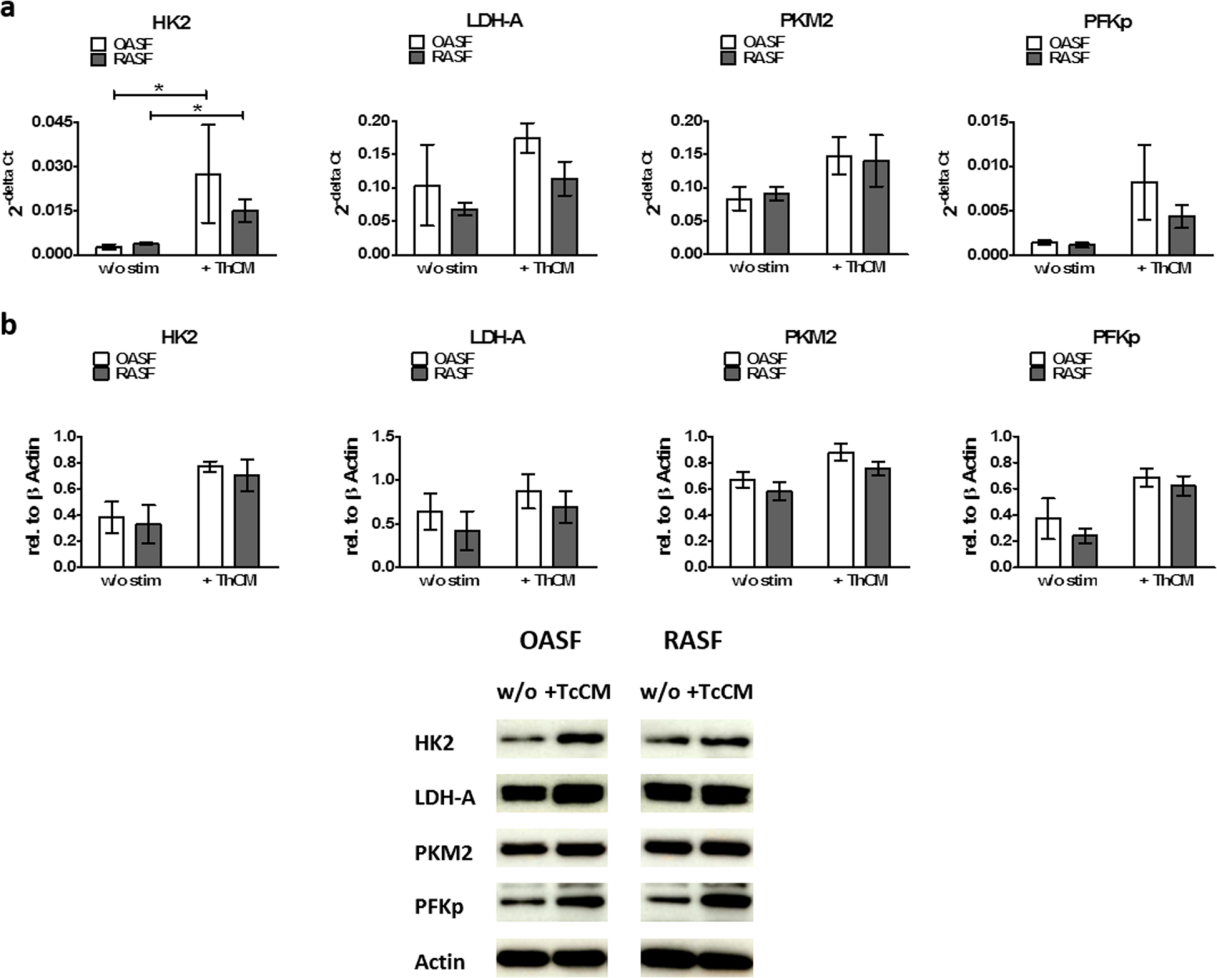
Stimulation of SF by Th cells results in an enhanced expression of glycolytic enzymes. OASF and RASF were cultured under resting conditions or under stimulation by ThCM and cells were harvested on d4. **a** The mRNA expression of HK2, LDH-A, PKM2 and PFKp were determined by RT-PCR. Each bar indicates the mRNA expression of the corresponding gene as 2^−deltaCT^ using β-actin mRNA expression as reference (*n* = 6). **b** Protein levels of HK2, LDH-A, PKM2 and PFKp were quantified by western blot. β-Actin was used as a loading control. The depictions are representative examples from one of three independent experiments. HK2, hexokinase 2; LDH-A, lactate dehydrogenase A; PKM2, pyruvate kinase M2; PFKp, phosphofructokinase p. Data are presented as the mean ± SEM. *p < 0.05, Mann-Whitney *U* test and Wilcoxon signed-rank test

Since RASF displayed increased baseline levels of glycolysis under resting conditions when compared to OASF and stimulation by ThCM boosted the glycolytic activity of both OASF and RASF, to further elucidate the role of glycolytic key regulators in the enhanced glycolytic activity of RASF and the shift in glucose metabolism towards glycolysis upon stimulation by Th cells, we quantified the expression of HKII, PFKp, PKM2 and LDH-A in OASF and RASF under resting conditions and under stimulation by ThCM. Even though there was more aerobic glycolysis in resting RASF than in OASF, we did not find any differences in the mRNA and protein expression of the analysed glycolytic enzymes between resting OASF and RASF (Fig. 4a, b). Stimulation with ThCM resulted in a significant increase in the expression of HK2 mRNA by both RASF and OASF compared to unstimulated conditions. For PFKp, PKM2 and LDH-A, a tendency towards higher mRNA expression upon stimulation by ThCM could be observed (Fig. 4a). Comparable results were obtained on the protein level by western blot and immunofluorescence analysis (Fig. 4b and Additional file 6 Fig. S6).

### Targeting particular cytokines by biologics is not sufficient to reduce the Th cell-stimulated glycolysis in SF

**Fig. 5:**
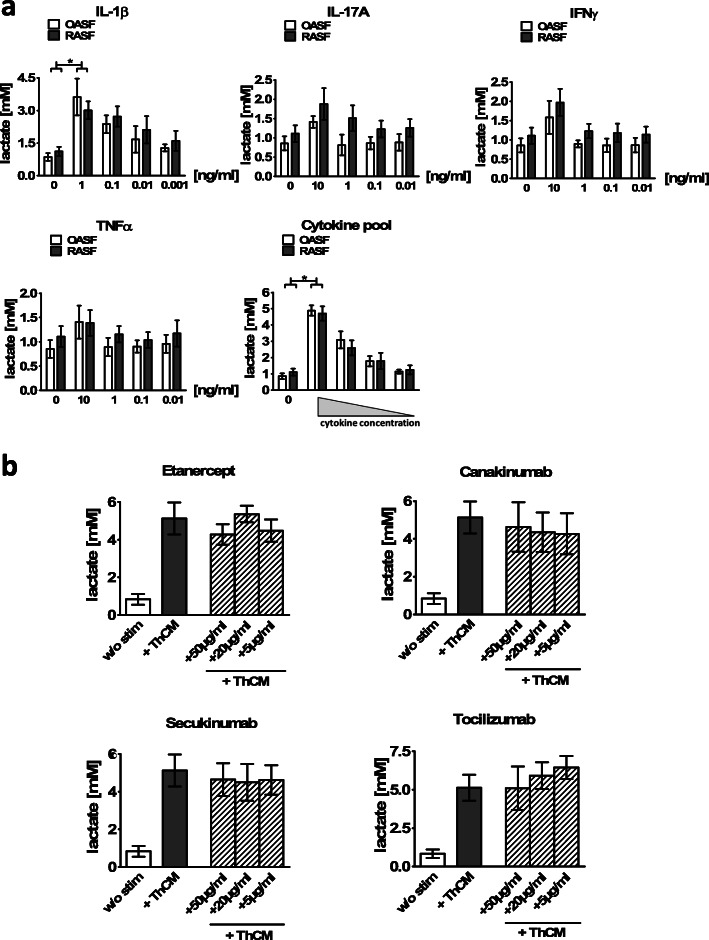
Effects of stimulation with cytokines and blocking of cytokines on the glucose metabolism of SF. **a** OASF and RASF were stimulated with different concentrations of IL-1β, IL-17A, TNFα and IFNγ, either individually or with all four cytokines. After 4 days of culture, the supernatants were harvested, and lactate concentrations were measured by ^1^H NMR spectroscopy (*n* = 6). **b** OASF were cultured under resting conditions or with stimulation by ThCM in the presence or absence of anti-TNFα (etanercept), anti-IL-6 receptor (tocilizumab), anti-IL-17A (secukinumab) and anti-IL-1β (canakinumab) at the three given concentrations. Lactate concentrations were quantified on d4 (*n* = 4). Data are shown as the mean ± SEM. *p < 0.05, Mann-Whitney U test and Wilcoxon signed-rank test

Our data presented above showed that both the production of pro-inflammatory cytokines and a metabolic shift towards aerobic glycolysis were induced in SF by ThCM by soluble factors in a cell contact-independent way. We next investigated whether cytokines known to play key roles in the pathogenesis of RA and to have the capacity to activate SF, namely TNFα, IL-1β and IL-17A, and IFNγ, which we found in highest concentrations in ThCM, are known to play a role in the pathogenesis of RA or other rheumatic diseases, alone or in combination, are able to induce the production of lactate by SF. Therefore, OASF and RASF were incubated with different concentrations of recombinant TNFα, IL-1β, IL-17A and IFNγ, either individually or with all four cytokines. On day 4, the lactate production by the stimulated SF was quantified. As presented in Fig. 5a, all individual cytokines induced a trend towards an enhanced lactate production by both OASF and RASF, at least at the highest concentrations tested. However, only IL1-β had a significant and dose-dependent glycolysis-promoting effect on OASF and RASF. In addition, simultaneous stimulation by all four cytokines together revealed a synergistic effect resulting in a higher increase in lactate production by both OASF and RASF than the one observed with the individual cytokines (Fig. 5a).

Neutralizing cytokines or cytokine receptors by specific antibodies have proven to be greatly effective in the treatment of rheumatic diseases. In order to determine whether such biologics could abrogate the upregulation of aerobic glycolysis in Th cell-stimulated SF, OASF were incubated with ThCM in the presence of different concentrations of etanercept (anti-TNFα), tocilizumab (anti-IL-6-receptor), secukinumab (anti-IL-17A) or canakinumab (anti-IL-1β), and the production of lactate was analysed on day 4. As depicted in Fig. 5b, none of the biologics caused a significant reduction in lactate production by OASF stimulated with ThCM. Thus, pro-inflammatory cytokines, especially IL-1β, can induce aerobic glycolysis in SF. However, targeting only one cytokine or cytokine receptor by the biologics was not sufficient to significantly affect the Th cell-mediated metabolic switch in SF.

### Inhibition of JAKs as well as glycolytic enzymes both efficiently block the Th cell-mediated switch towards a glycolytic and inflammatory phenotype in SF

**Fig. 6:**
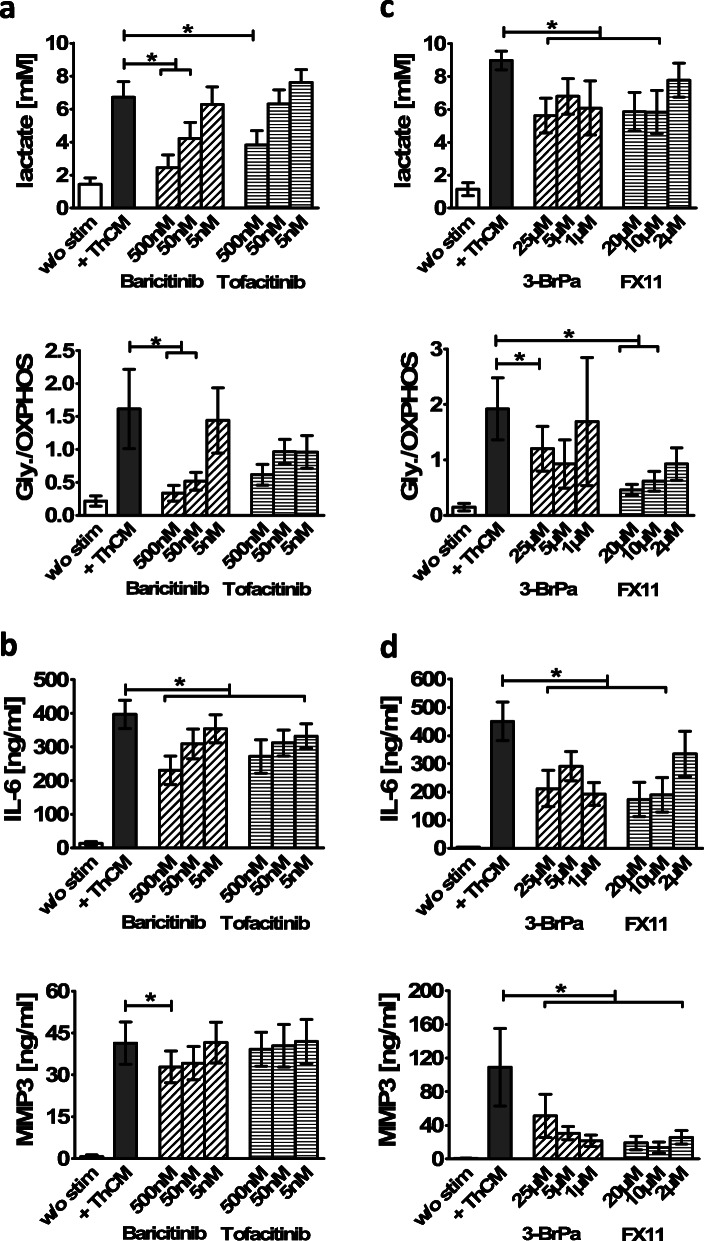
Targeting JAKs or glycolytic enzymes inhibits the development of a glycolytic and pro-inflammatory SF phenotype. OASF were cultured with or without stimulation by ThCM in the presence or absence of different concentrations of baricitinib, tofacitinib or the glycolytic inhibitors 3-BrPa and FX11. After 4 days of culture, supernatants were harvested, and the lactate concentrations and the ratio of glycolytic to oxidative glucose metabolism (**a**, **c**) as well as the secretion of IL-6 and MMP3 (**b** and **d**) were determined (*n* = 7). Results are presented as the mean ± SEM. *p < 0.05, Wilcoxon signed-rank test. 3-BrPa, 3-bromopyruvate

Since the biologics targeting single cytokines were inefficient in lowering the stimulatory effect of ThCM on SF’s glycolytic metabolism, we next tested whether blocking intracellular cytokine signalling through the JAK pathway could more efficiently block Th cell-mediated SF activation. Most of the cytokines which we detected in ThCM are known to induce cytokine receptor signalling via the JAK-STAT pathway, and stimulation by soluble mediators released by activated Th cells strongly induced phosphorylation of STAT in OASF and RASF [28]. For this reason, SF were stimulated with ThCM in the presence or absence of the JAKi baricitinib or tofacitinib. Remarkably, both baricitinib and tofacitinib significantly diminished the production of lactate by SF stimulated by ThCM in a dose-dependent manner (Fig. 6a). Additionally, baricitinib significantly abrogated the shift from oxidative to glycolytic glucose metabolism in SF. Importantly, in parallel to their effect on the glycolytic rate, both JAKi significantly reduced the secretion of IL-6 by Th cell-stimulated SF (Fig. 6b). Secretion of MMP3 was less affected by JAKi, though baricitinib significantly reduced MMP3 expression by SF at the highest concentration tested (500 nM) (Fig. 6b).

Finally, we hypothesized that the shift towards glycolytic glucose metabolism and the induction of an inflammatory, matrix-degrading SF phenotype are not independent processes induced in SF by Th cells, but rather that the upregulation of aerobic glycolysis represents a *conditio* sine qua non for the adaptation of a pro-inflammatory phenotype by SF. To test this hypothesis, we blocked glycolysis in Th cell-stimulated SF by adding either 3-BrPA, an inhibitor of HK2, or FX11, an inhibitor of LDH-A, and analysed the effects on lactate, IL-6 and MMP3 production. As expected, both inhibitors significantly diminished the production of lactate and the ratio of glycolytic versus oxidative glucose metabolism compared to Th cell-stimulated SF in the absence of inhibitors (Fig. 6c). Moreover, both 3-BrPA and FX11 curbed the pro-inflammatory phenotype by strongly reducing the secretion of IL-6 and MMP3 by SF stimulated with ThCM (Fig. 6d). The viability of the SF was not affected by the 3-BrPa and FX11 concentrations used in these experiments (Additional file 7 Fig. S7). Thus, inhibition of glycolysis in Th cell-stimulated SF significantly impeded the development of an inflammatory phenotype. Importantly, both glycolytic inhibitors as well as the JAKi potently limited the Th cell-mediated stimulation of aerobic glycolysis and of an inflammatory, matrix-degrading phenotype in SF.

## Discussion

Recently, several studies have reported metabolic changes in immune and resident synovial cells as well as systemic metabolic alterations in RA patients [11, 14, 19–21, 23, 36, 37]. Dysregulated bioenergetics with mitochondrial dysfunction and a switch from oxidative towards glycolytic glucose metabolism has been shown to be a determinant for inflammatory and aggressive functions of SF in RA [19, 20, 38]. Although this increase in glycolysis is considered to be crucial in the pathogenesis of RA, very little is known about the influence of immune cells on the glucose metabolism of SF. In this study, we investigated the effect of Th cells on the glucose metabolism of both RASF, with their intrinsically activated phenotype, and OASF and the effects of targeting cytokines, cytokine receptor signalling and glycolytic enzymes on the phenotype of Th cell-stimulated SF.

First, we demonstrated that quiescent RASF had a significantly higher glycolytic activity and lactate production compared to OASF. The elevated glycolytic activity was accompanied by a significantly higher secretion of IL-6 and MMP3 by RASF compared to OASF under resting conditions. These results confirm previous studies reporting a higher glycolytic activity in quiescent RASF compared to OASF [19, 39]. Garcia-Carbonell and colleagues found that the higher baseline mitochondrial respiration rate in RASF compared to OASF correlated with a higher expression of the glucose transporter GLUT1 and of HK2 in RASF [19]. In our study, a significant difference in the expression of HK2 or of the glycolytic enzymes LDH-A, PKM2 and PFKp between quiescent RASF and OASF could not be detected, neither at the mRNA level nor the protein level, despite the significantly higher lactate secretion observed in resting RASF. Stimulation with LPS has been shown to induce a metabolic shift towards aerobic glycolysis and increased lactate secretion in both OASF and RASF, while stimulation with platelet-derived growth factor (PDGF) induced a significantly lower glycolytic response in OASF compared to RASF, demonstrating that the metabolic response of SF strongly depends on the activating stimulus [19]. Here, we have shown that stimulation with culture supernatants of activated Th cells dramatically changed the metabolic profile of both RASF and OASF compared to resting conditions by significantly increasing the activity of aerobic glycolysis and HK2 expression. Importantly, under stimulation by Th cells, OASF showed a similarly high glycolytic activity, lactate production and expression of glycolytic enzymes as RASF. In addition, significant differences could not be detected in the secretion of IL-6, IL-8 and MMP3 between Th cell-stimulated OASF and RASF. Thus, activated Th cells have the capability to induce a pro-inflammatory, RA-like phenotype with high glycolytic activity even in OASF. As we have shown before, interaction with activated lymphocytes can convert OASF into SF with a pro-inflammatory and cartilage-invading RA-like phenotype [27, 28, 40]. An amplification of the glycolytic glucose metabolism is therefore likely to play an important role in the induction of an aggressive SF phenotype.

Enhanced migration in response to pro-inflammatory stimuli like PDGF, Toll-like receptor 2 activation, IL-1β or TNFα has been described for RASF as part of their aggressive phenotype [19, 38, 41, 42]. An enhanced invasiveness has also been shown in transwell matrigel assays for RASF in response to soluble mediators released by Th cells [26]. In contrast to these findings, we demonstrate here that the migratory capacities of OASF and RASF are significantly reduced under stimulation with ThCM and also under stimulation with inflammatory cytokines, especially when applied in combination. Reduced wound healing migration of fibroblasts in response to IFNγ or TNFα has been described before [43–45]. Within the synovium, a diminished migration of SF, e.g. towards cartilage, would be beneficial, and therefore, it may possibly represent another anti-inflammatory mechanism induced by the interaction between Th cells and SF [27]. Further studies would be needed to confirm this hypothesis.

Interestingly, a pro-inflammatory memory has been described for SF that have been primed by an inflammatory stimulus [35]. A pathogenic memory of their aggressive phenotype is well known for RASF [4]. Crowley et al. found that even SF from non-inflamed joints, but not dermal fibroblasts, showed an augmented response to an inflammatory stimulus after a second challenge compared to a primary response [35]. In their study, the inflammatory memory of SF was only transient. However, the effect of chronic stimulation has not been investigated. Here, we have demonstrated that chronic stimulation of SF over 12 days led to a significantly enhanced “inflammatory memory” compared to a single or even a second stimulation. Importantly, when re-stimulated after a resting period, the chronically stimulated SF not only showed a higher IL-6 and MMP3 expression, but also a significantly augmented lactate production compared to the other groups. Thus, repeated stimulation of SF in an inflammatory micro-environment not only results in the development of an “inflammatory memory”, but also of a “glycolytic memory”. Continuing studies may reveal if such a metabolic memory plays a role in the switch from an acute resolving joint inflammation to a chronic persistent joint inflammation as seen in RA.

The therapeutic targeting of individual cytokines or cytokine receptors by biologics is effective in the treatment of RA. We therefore tested the potential of biologics used in RA therapy for blocking the glycolytic activity of SF stimulated by Th cells. However, we showed that none of the tested biologics, neutralizing TNFα, IL1β, IL-17A or IL-6R, could reduce the amount of lactate secreted by Th cell-stimulated SF. This observation fits our finding that lactate production is more efficiently induced by stimulation of SF with a combination of different cytokines than by individual cytokines. A synergistic effect of cytokines in the induction of an inflammatory, aggressive phenotype by SF has been described before [46, 47].

The inhibition of JAK has proven to be greatly effective in the treatment of RA, even in patients who are refractory to treatment with biologics. The pivotal advantage of JAKi over cytokine-neutralizing biologics relies on their capacity to simultaneously block the intracellular signalling of multiple cytokines. We therefore hypothesized that JAKi would be more efficient in blocking the shift towards glycolytic metabolism as well as the aggressive phenotype in Th cell-stimulated SF compared to biologics. Indeed, JAKi baricitinib and tofacitinib both significantly reduced the production of lactate in OASF stimulated by Th cells and both lowered the ratio of aerobic glycolysis to oxidative phosphorylation, with baricitinib showing a higher efficiency than tofacitinib. On the other hand, both baricitinib and tofacitinib significantly reduced the secretion of IL-6 by SF, suggesting a direct link between JAK/STAT signalling and the regulation of inflammatory as well as metabolic pathways. Activation of JAK/STAT3 signalling in RASF has been shown to result in the enhanced expression of key glycolytic enzymes and augmented aerobic glycolysis [48, 49]. Moreover, activation of hypoxia-inducible factor (HIF)1α by pSTAT3 can trigger a vicious glycolytic, inflammatory cycle since HIF1α stimulates the transcription of PKM2, HK2 and other glycolytic enzymes and the kinase PKM2 directly phosphorylates STAT3 [50]. Interestingly, lactate has been shown to stabilize HIF1α [51]. Treatment of RASF or of RA ex vivo synovial explants with tofacitinib decreased HIF1α, HK2 and LDH-A expression and glycolytic activity [48]. In parallel, tofacitinib significantly inhibited the secretion of IL-6, confirming our results. Altogether, JAK/STAT signalling induces the expression of glycolytic enzymes and triggers a shift in glucose metabolism towards aerobic glycolysis. Inhibition of JAKs prevents the upregulation of glycolysis and the pro-inflammatory phenotype of SF.

Direct therapeutic targeting of glycolytic enzymes and manipulation of the cell metabolism has recently been considered to offer new avenues in the treatment of rheumatic diseases [52, 53]. Genetic ablation of HK2 has been shown to attenuate the severity of inflammatory arthritis and reduce bone and cartilage damage in mouse models [54]. We used 3-BrPa, which antagonizes HK2, and FX11, an inhibitor of LDH-A, to block glycolysis in Th cell-stimulated SF. Both inhibitors significantly reduced lactate production and the glycolytic rate in SF. In addition, both glycolytic inhibitors strongly downregulated the secretion of IL-6 and MMP3 by SF. Hence, our data indicate that a glycolytic metabolism is necessary for the initiation of a pro-inflammatory phenotype in OASF.

In a recent study, Petrasca and colleagues demonstrated that Th cells promoted the aggressive phenotype in RASF by boosting glycolysis—confirmed here with our data—and this effect could be reversed by 2-deoxy-d-glucose (2-DG) or by the AMP analogue 5-aminoimidazole-4-carboxamide ribonucleotide [26]. An abrogation of the aggressive phenotype of RASF by 2-DG, a non-metabolizable glucose analogue which blocks glycolysis downstream of HK2, has also been described by others [19], and 2-DG has been shown to be effective in reducing the severity of arthritis in a mouse model [55]. In the same manner, treatment with 3-BrPa also significantly reduced the severity of arthritis in mouse models [19, 56]. Inhibition of another critical enzyme in the glycolytic pathway, 6-phosphofructo-2-kinase/fructose-2,6-biphosphatase, also reduced the aggressive phenotype of RASF and attenuated joint inflammation in an RA mouse model [57]. LDH-A is the most downstream enzyme in the glycolytic breakdown of glucose, catalyzing the conversion of pyruvate to lactate. Since pyruvate can also be metabolized by the Krebs cycle, therapeutic targeting of LDH-A may cause minor side effects compared to the inhibition of other glycolytic enzymes. For the treatment of cancer, the blocking LDH-A by the specific inhibitor FX11 has already demonstrated preclinical efficacy [58–61]. However, clinical trials are still missing. Recently, we demonstrated that LDH-A is overexpressed in CD8^+^ T cells of RA patients and that inhibition of LDH-A with FX11 reduced lipogenesis, migration, proliferation and effector functions of RA CD8^+^ T cells [62]. In the present study, we have shown for the first time that treatment with FX11 not only significantly lowered lactate production and the glycolytic rate in SF stimulated by Th cells, but also strongly reduced the inflammatory, matrix-degrading SF phenotype. Importantly, our results demonstrate that the complete glycolytic pathway with lactate, and not pyruvate, as its end product is required for Th cell-stimulated SF to develop an inflammatory phenotype. Most likely this is due to the fact that a maintenance of nicotinamide adenine dinucleotide (NAD^+^) levels is crucial for ATP production and mitochondrial homeostasis. By the glycolytic conversion of glucose to pyruvate, NAD^+^ is reversibly reduced to NADH. The final conversion of pyruvate to lactate by LDH regenerates NAD^+^ from NADH and thereby maintains the balance between NAD^+^ and NADH [63, 64].

All-in-all, targeting glycolytic glucose metabolism clearly bears potential as a therapeutic strategy in RA. A decisive factor will be the level of specificity for targeting only pathogenic cells by glycolysis inhibitors and thereby minimizing side effects. Our data reveals that JAKi are as efficient as glycolytic enzyme inhibitors in suppressing glycolytic activity as well as the inflammatory phenotype of SF activated by Th cells. Baricitinib and tofacitinib are already approved for RA therapy. However, it may be that JAKi are not effective in abrogating glycolytic and destructive properties of SF in late, chronic stages of RA when SF maintain their aggressive phenotype independent of an inflammatory micro-environment. In this case, glycolytic inhibitors could present a useful alternative.

## Conclusions

The induction of a pro-inflammatory phenotype in SF mediated by chronic stimulation with Th cells is functionally linked to a metabolic shift towards aerobic glycolysis in SF. While the blocking of cytokines or cytokine receptors by biologics was not sufficient to limit the glycolytic activity in Th cell-stimulated SF, targeting JAKs or glycolytic enzymes both efficiently inhibited the switch towards a glycolytic and inflammatory phenotype in SF. Thus, in addition to the JAKi baricitinib and tofacitinib, therapeutic manipulation of the glucose metabolism of SF by glycolytic inhibitors like 3-BrPa and FX11 holds potential as a treatment strategy for inflammatory arthritis independent of a systemic immunosuppression.

## Supplementary Information

**Additional file 1 **: **Figure S1.** Soluble mediators released by the Th cells of RA patients and those of healthy individuals induce similar rates of lactate secretion in SF. OASF and RASF were cultured in the presence of [U-^13^C]-glucose either under resting conditions or stimulated by conditioned culture media of activated Th cells isolated either from normal healthy donors (ThCM NHD) or from RA patients (ThCM RA). After 4 days, [U-^13^C]-lactate concentrations in the culture supernatants were determined by ^1^H NMR spectroscopy. Results are presented as the mean±SEM, *n*=6. (*) *p*<0.05, Wilcoxon signed-rank test. w/o stim: without stimulation.

**Additional file 2 **: **Figure S2.** Dose-dependent induction of lactate production in SF by soluble mediators released by Th cells. OASF (n=6) and RASF (n=6) were cultured in the presence of [U-^13^C]-glucose either under resting conditions or stimulated by different dilutions of ThCM. [U-^13^C]-lactate concentrations in the culture supernatants were determined after 4 days of culture using ^1^H NMR spectroscopy. Results are presented as the mean±SEM. (*) *p*<0.05, (**) *p*<0.01, Wilcoxon signed rank test. w/o stim: without stimulation.

**Additional file 3 **: **Figure S3.** Quantification of the cytokine content of ThCM. CD4^+^ Th cells isolated from the peripheral blood of RA patients were stimulated by anti-CD3/ anti-CD28 antibodies for 4 days. Cytokines released by Th cells into the culture medium were quantified by flow cytometry using a LEGENDplex flow assay kit (BioLegend). Results are presented as the mean±SEM in logarithmic scale; *n*=15.

**Additional file 4 **: **Figure S4.** The influence of a hypoxic microenvironment on the glucose metabolism of SF. OASF and RASF were cultured for 4 days under hypoxic conditions (3% O_2_) either in the presence or absence of ThCM. **a** The amount of secreted [U-^13^C]-lactate and the ratio of [U-^13^C]-glucose metabolized by glycolysis (Gly) to that metabolized by oxidative phosphorylation (OXPHOS) was determined in SF culture supernatants using ^1^H NMR spectroscopy (*n*=12). **b** SF were stimulated by different dilutions of ThCM under hypoxia. [U-^13^C]-lactate concentrations were determined after 4 days of culture (*n*=6). Results are presented as the mean±SEM. (*) p<0.05, (**) p<0.01, Mann-Whitney U test and Wilcoxon signed-rank test. w/o stim: without stimulation; ThCM: conditioned culture media of activated Th cells.

**Additional file 5 **: **Figure S5.** Reduced migration of SF in response to stimulation by mediators released by Th cells. The migratory capacities of OASF and RASF were analysed by a wound healing scratch assay. After inducing a scratch into confluent monolayers of RASF or OASF, cells were cultured in the presence or absence of ThCM or stimulated by IFNγ, TNFα or IL-17A (10 ng/ml) or by a mixture of all three cytokines. Wound closure was monitored by light microscopy and cell migration was determined by comparing the gap width on days 0 and 3. **a** Representative microscopic images of the scratched wound region on days 0 and 3. **b** The diagram displays the migration of OASF (*n*=3) and RASF (n=3) under stimulation by ThCM or cytokines as the percentage of wound gap closure, presented as the mean±SEM.

**Additional file 6 **: **Figure S6.** Immunofluorescence staining of resting SF and Th cell-stimulated SF for HK2 and PKM2. OASF and RASF were cultured under resting conditions or under stimulation by ThCM on glass coverslips. On day 4, cells were stained with antibodies against HK2 and PKM2 and a Cy3-labelled secondary antibody (red staining). Cell nuclei were stained with DAPI (blue staining). The depictions are taken from one of four independent experiments and are valid representative. HK2 = hexokinase 2; PKM2 = pyruvate kinase M2; ab = antibody.

**Additional file 7 **: **Figure S7.** Viability of Th cell-stimulated SF treated with the glycolytic inhibitors 3-BrPa and FX11. SF were stimulated with ThCM in the presence or absence of different concentrations of 3-BrPa and FX11. After 4 days of culture, cells were harvested, stained with Annexin V (AxV) and propidium iodide (PI) and cell viability was determined by flow cytometry. Treatment of SF with 2μM staurosporin within the last 24 hours of culture served as a positive control for cell apoptosis. Data are presented as the mean±SEM, *n*=6. (*) *p*<0.05, Wilcoxon signed-rank test. 3-BrPa = 3-bromopyruvate.

## Data Availability

All data generated or analysed during this study are included in this published article and its supplementary information files.

## Abbreviations

3-BrPa: 3-Bromopyruvate
DAPI: 4′,6-Diamidino-2-phenylindole
2-DG: 2-Deoxy-d-glucose
ELISA: Enzyme-linked immunosorbent assay
^1^H NMR: Proton nuclear magnetic resonance spectroscopy
HIF: Hypoxia-inducible factor
HK: Hexokinase
IFN: Interferon
IL: Interleukin
JAKi: Janus kinase inhibitor
JAKs: Janus kinases
LDH: Lactate dehydrogenase
MMP: Matrix metalloproteinase
NHD: Normal healthy donor
OA: Osteoarthritis
PDGF: Platelet-derived growth factor
PFK: Phosphofructokinase
PKM2: Pyruvate kinase M2
RA: Rheumatoid arthritis
SF: Synovial fibroblasts
Th: T helper
ThCM: Conditioned media of activated Th cells
TNF: Tumour necrosis factor
