# Supplementary figures and images for: Increase of aerobic glycolysis mediated by activated T helper cells drives synovial fibroblasts towards an inflammatory phenotype: new targets for therapy?
Source: Arthritis Res Ther. 2021 Feb 15;23:56. doi: 10.1186/s13075-021-02437-7 (PMC7883459; doi:10.1186/s13075-021-02437-7)

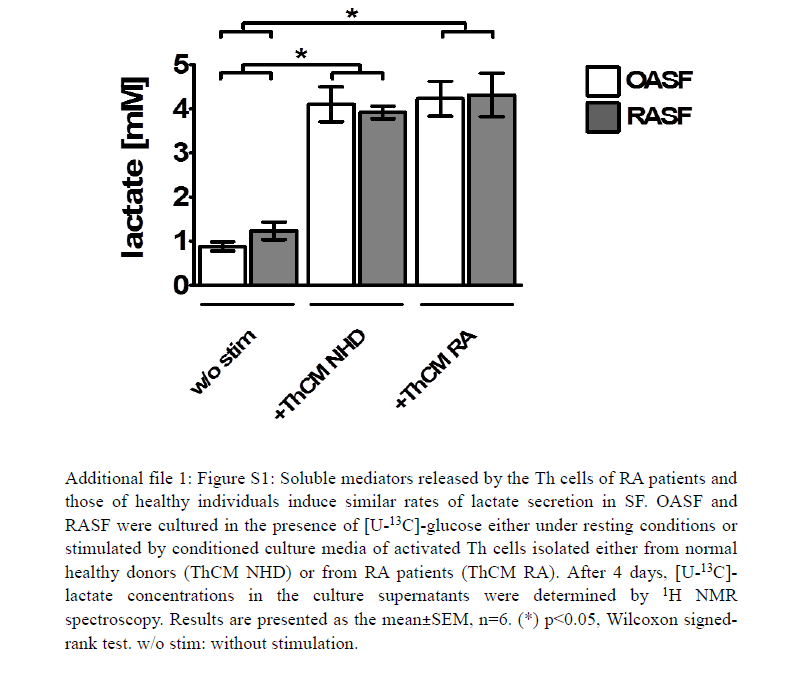

Supplement: Supplementary file 1 — Additional file 1 : Figure S1. Soluble mediators released by the Th cells of RA patients and those of healthy individuals induce similar rates of lactate secretion in SF. OASF and RASF were cultured in the presence of [U-13C]-glucose either under resting conditions or stimulated by conditioned culture media of activated Th cells isolated either from normal healthy donors (ThCM NHD) or from RA patients (ThCM RA). After 4 days, [U-13C]-lactate concentrations in the culture supernatants were determined by 1H NMR spectroscopy. Results are presented as the mean±SEM, n=6. (*) p<0.05, Wilcoxon signed-rank test. w/o stim: without stimulation. [file 13075_2021_2437_MOESM1_ESM.png]

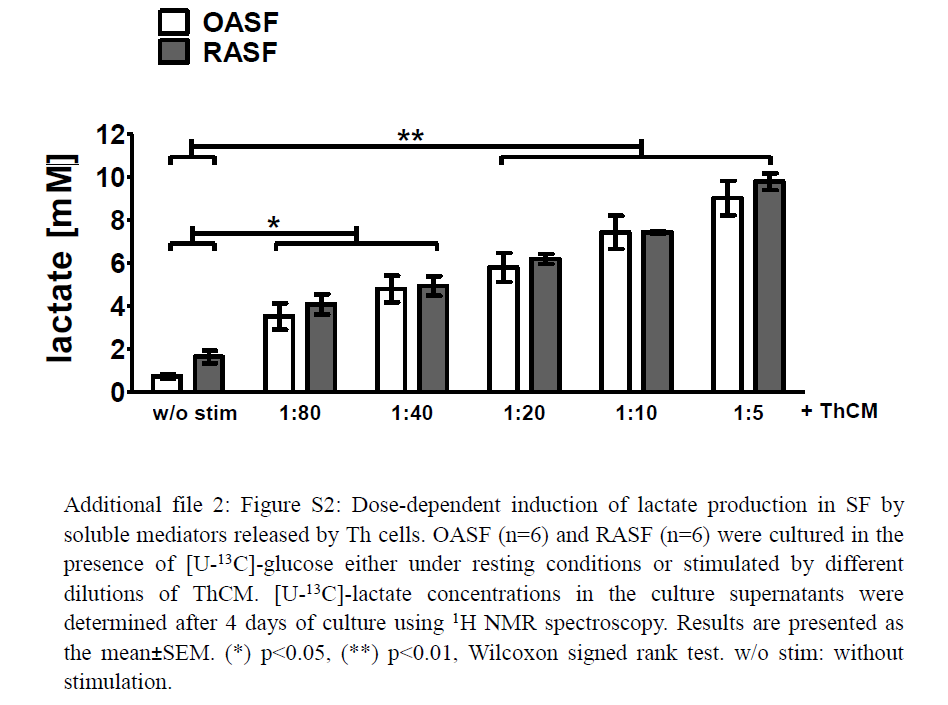

Supplement: Supplementary file 2 — Additional file 2 : Figure S2. Dose-dependent induction of lactate production in SF by soluble mediators released by Th cells. OASF (n=6) and RASF (n=6) were cultured in the presence of [U-13C]-glucose either under resting conditions or stimulated by different dilutions of ThCM. [U-13C]-lactate concentrations in the culture supernatants were determined after 4 days of culture using 1H NMR spectroscopy. Results are presented as the mean±SEM. (*) p<0.05, (**) p<0.01, Wilcoxon signed rank test. w/o stim: without stimulation. [file 13075_2021_2437_MOESM2_ESM.png]

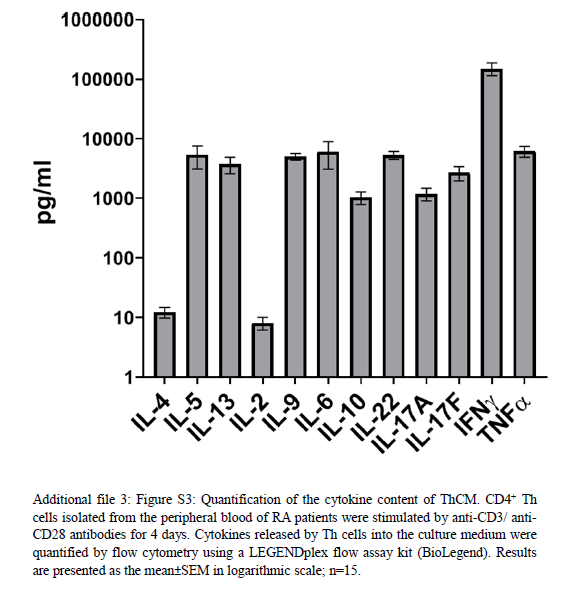

Supplement: Supplementary file 3 — Additional file 3 : Figure S3. Quantification of the cytokine content of ThCM. CD4+ Th cells isolated from the peripheral blood of RA patients were stimulated by anti-CD3/ anti-CD28 antibodies for 4 days. Cytokines released by Th cells into the culture medium were quantified by flow cytometry using a LEGENDplex flow assay kit (BioLegend). Results are presented as the mean±SEM in logarithmic scale; n=15. [file 13075_2021_2437_MOESM3_ESM.png]

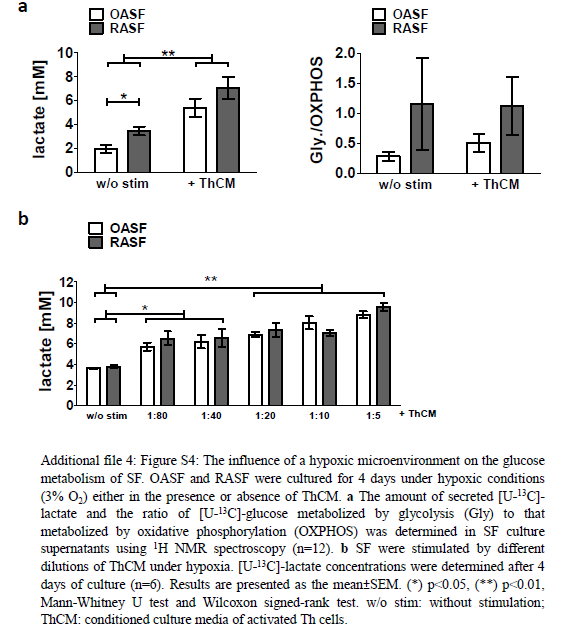

Supplement: Supplementary file 4 — Additional file 4 : Figure S4. The influence of a hypoxic microenvironment on the glucose metabolism of SF. OASF and RASF were cultured for 4 days under hypoxic conditions (3% O2) either in the presence or absence of ThCM. a The amount of secreted [U-13C]-lactate and the ratio of [U-13C]-glucose metabolized by glycolysis (Gly) to that metabolized by oxidative phosphorylation (OXPHOS) was determined in SF culture supernatants using 1H NMR spectroscopy (n=12). b SF were stimulated by different dilutions of ThCM under hypoxia. [U-13C]-lactate concentrations were determined after 4 days of culture (n=6). Results are presented as the mean±SEM. (*) p<0.05, (**) p<0.01, Mann-Whitney U test and Wilcoxon signed-rank test. w/o stim: without stimulation; ThCM: conditioned culture media of activated Th cells. [file 13075_2021_2437_MOESM4_ESM.png]

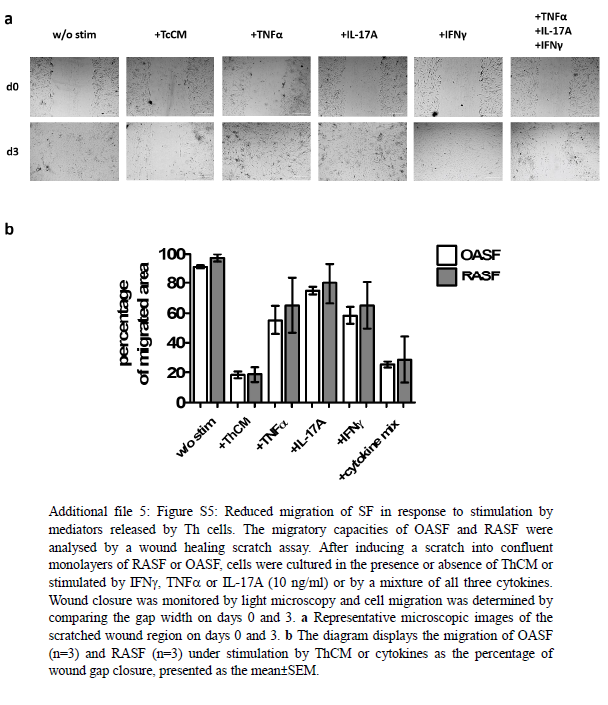

Supplement: Supplementary file 5 — Additional file 5 : Figure S5. Reduced migration of SF in response to stimulation by mediators released by Th cells. The migratory capacities of OASF and RASF were analysed by a wound healing scratch assay. After inducing a scratch into confluent monolayers of RASF or OASF, cells were cultured in the presence or absence of ThCM or stimulated by IFNγ, TNFα or IL-17A (10 ng/ml) or by a mixture of all three cytokines. Wound closure was monitored by light microscopy and cell migration was determined by comparing the gap width on days 0 and 3. a Representative microscopic images of the scratched wound region on days 0 and 3. b The diagram displays the migration of OASF (n=3) and RASF (n=3) under stimulation by ThCM or cytokines as the percentage of wound gap closure, presented as the mean±SEM. [file 13075_2021_2437_MOESM5_ESM.png]

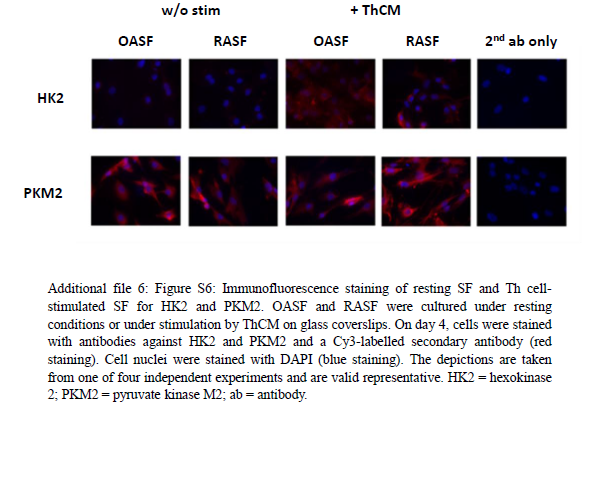

Supplement: Supplementary file 6 — Additional file 6 : Figure S6. Immunofluorescence staining of resting SF and Th cell-stimulated SF for HK2 and PKM2. OASF and RASF were cultured under resting conditions or under stimulation by ThCM on glass coverslips. On day 4, cells were stained with antibodies against HK2 and PKM2 and a Cy3-labelled secondary antibody (red staining). Cell nuclei were stained with DAPI (blue staining). The depictions are taken from one of four independent experiments and are valid representative. HK2 = hexokinase 2; PKM2 = pyruvate kinase M2; ab = antibody. [file 13075_2021_2437_MOESM6_ESM.png]

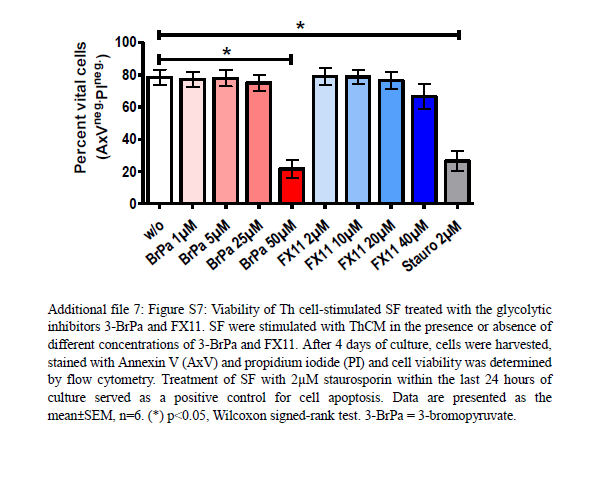

Supplement: Supplementary file 7 — Additional file 7 : Figure S7. Viability of Th cell-stimulated SF treated with the glycolytic inhibitors 3-BrPa and FX11. SF were stimulated with ThCM in the presence or absence of different concentrations of 3-BrPa and FX11. After 4 days of culture, cells were harvested, stained with Annexin V (AxV) and propidium iodide (PI) and cell viability was determined by flow cytometry. Treatment of SF with 2μM staurosporin within the last 24 hours of culture served as a positive control for cell apoptosis. Data are presented as the mean±SEM, n=6. (*) p<0.05, Wilcoxon signed-rank test. 3-BrPa = 3-bromopyruvate. [file 13075_2021_2437_MOESM7_ESM.png]
